# Forecasting the future of smart hospitals: findings from a real-time delphi study

**DOI:** 10.1186/s12913-024-11895-z

**Published:** 2024-11-18

**Authors:** Florian Jovy-Klein, Susan Stead, Torsten Oliver Salge, Jil Sander, Anke Diehl, David Antons

**Affiliations:** 1 RWTH Aachen University, Institute for Technology and Innovation Management, Kackerstr. 7, Aachen, 52072 Germany; 2 University Medicine Essen, Digital Transformation Unit, Hufelandstraße 55, Essen, 45147 Germany; 3 Institute for Entrepreneurship, University of Bonn, Meckenheimer Allee 174, 53115 Bonn, Germany

**Keywords:** Smart hospital, Forecasting, Real-time Delphi, Artificial Intelligence, Human-centeredness, Ecosystems

## Abstract

**Background:**

In concert with other digital technologies, artificial intelligence (AI) is shaping the vision of smart hospitals. The transformation into smart hospitals, however, is all but trivial due to the lack of financial and human resources, digital skills, and supporting policies. Thus, the extent to which the vision of smart hospitals will eventually become reality is uncertain. In this context, our study provides a multidimensional conceptualization of the immediate future of smart hospitals to 2042.

**Methods:**

This study employs an iterative mixed-methods approach, including expert workshops and a Delphi study. We conducted a real-time Delphi study to forecast the evolution of smart hospitals in 5-year steps from 2027 to 2042. A total of 39 experts in healthcare, artificial intelligence, and management participated.

**Results:**

Our understanding of a technology-enabled smart hospital in this study includes four dimensions: artificial intelligence (AI), sustainability, ecosystems, and human-centeredness. Our findings underscore the critical need to address the shortage of hospital staff and general practitioners that models predict will peak by 2032. Additionally, our results show a significant shift to individualized medicine and home care. This shift indicates that smart hospitals are expected to leverage AI and digital technologies to tailor care to each patient. Furthermore, the roles and responsibilities of hospital staff will undergo significant changes. Healthcare personnel will have to adapt to new technologies that facilitate more efficient workflows and improve patient engagement in evolving healthcare environments. The results of our study suggest a shift in care to individualized medicine and home care, with corresponding changes in the roles and responsibilities of hospital staff who will employ new technologies.

**Conclusions:**

The findings from our real-time Delphi study suggest that the vision of smart hospitals is gradually becoming reality over the next 20 years. Advancements in artificial intelligence should enhance operational efficiency and patient-centric care, while facilitating the integration of sustainability practices and fostering collaborative ecosystems. However, addressing challenges such as staff shortages, ethical considerations, and the need for robust digital skills will be essential. A deep pool of expert healthcare practitioners, clear ethical guidelines, and robust digital skills are essential to fully realize this vision and ensure that smart hospitals can meet the evolving needs of healthcare delivery.

**Supplementary Information:**

The online version contains supplementary material available at 10.1186/s12913-024-11895-z.

## Introduction

Globally, the healthcare industry, particularly the hospital sector, faces an inflection point due to demographic and technological challenges. Labor shortages [1] and an aging population with an increasing prevalence of chronic and geriatric diseases [2] disrupt the provider-patient ratio. Platform ecosystems [3] emerging from other industries and tech companies arise to compete with traditional hospitals. Embracing the vision of smart hospitals addresses these challenges. Some hospitals have already begun this transformation [1, 4–6]. The vision of smart hospitals is fueled by powerful emerging digital technologies such as AI [7–10].

AI already plays an essential role in healthcare transformation, with care and business models changing [11] while technology’s impact on organizational processes increases [12]. However, uncertainty about the use of AI remains high. Substantial investment requirements, decentralized regional care structures, and unclear regulatory frameworks pose barriers to the adoption of AI and the smart hospital transformation process.

Germany is notable in this regard. The government understands the need for digital transformation. Officials also aim to pioneer the development of safe boundary conditions and implementation models that serve the entire sector. The “Act of the Hospital of the Future (KHZG)” [13] requires hospitals to comply with and implement digitalization measures that create boundaries for AI implementations and other digital solutions by 2025. To fulfill legal requirements and ensure the viability of the entire healthcare sector and of single hospital organizations, as well as prepare the industry and related organizations for the future, healthcare industry decision makers in need a clearer vision of smart hospitals. Unfortunately, extant literature paints an incomplete picture of how future hospitals may look, what the relevant dimensions of smart hospitals are, and what role AI will play. First, we identified a lack of clear definitions and frameworks for smart hospitals. Second, we identified a research gap regarding the challenges and influencing factors that shape the trend of transformation into smart hospitals. To illuminate this complex transformation process, we formulate the following research questions:

- RQ1) What characterizes a smart hospital and what are its most important dimensions?
- RQ2) What future challenges will the (German) healthcare sector face and how can AI address these challenges as hospitals transform into smart hospitals?

To answer these questions, we conducted a Delphi study with health and AI experts. We forecast the future of smart hospitals, the associated transformation challenges, and the broader healthcare context in which smart hospitals will be embedded. We focus on the period from 2027 to 2042. We hope that our insights will help decision-makers shape and implement the vision of smart hospitals.

### Conceptual background

Extant conceptualizations of smart hospitals are scarce and vary in detail and comprehensiveness. Kwon et al. 2020 center their conceptualization on information and communications technology. This includes aspects of digital services which provide energy and resource efficiency [14]. Holzinger et al. 2015, proposed that smart hospitals also need physical-digital ecosystems and human-centered designs to thrive [15]. This conception of a smart hospital as an intelligent, technology-driven yet human-centered system can be a fruitful starting point for our research. However, we see the need to further explicate the concept of smart hospitals and its constitutive dimensions. Below, we develop a conceptual framework that serves as the basis for the main Delphi study and our attempt to forecast the future of smart hospitals.

#### The artificial intelligence dimension of smart hospitals

Smart hospitals are seen as technologically advanced organizations that utilize AI and digital health data like eHealth, Telehealth, IoT solutions, robotics, high-speed communications [16, 17], and even blockchain [18].

Digital technologies that collect, process, and share data help enable AI usage [15, 16]. AI is already ubiquitous in computer science, research, and policy-making. However, it is still in its infancy in healthcare organizations [19], especially due to missing strategic guidance on implementation in healthcare processes [20]. AI promises to find patterns in data, extrapolate, interact, recognize objects, and detect illnesses [6]. The rise of computational power, storage capacity, and transmission speed (e.g. 5G) are paving the way for AI to become the powerhouse of future innovations and organizational efficiency. Besides being a specialized tool, AI will also be a key enabler for personalized medicine and systems medicine [21–23], as well as stakeholder-oriented healthcare [24]. As such, the use of AI will be a constitutive dimension of the concept and vision of smart hospitals.

Hurdles, however, are numerous. They include interoperability and regulatory [25] as well as data privacy concerns [26]. As health data often contain highly sensitive personal information, achieving privacy and trust that the technology will work error-free remain challenges for the future of smart hospitals [27].

#### The sustainability dimension of smart hospitals

Smart hospitals will also need to ensure their economic, social, and ecological sustainability. The UN Sustainable Development Goals, particularly the need for reliable and sustainable energy (SDG 7) and infrastructural resilience and sustainability (SDG 11) are of central importance for the future of hospitals [28]. Progress has been made, for instance, with sustainability in hospital food supplies and services [29]. Simultaneously, findings on enablers and inhibitors for sustainability actions in hospitals [30], show that digitalized processes can lead to decreased resource consumption compared to non-digital approaches. Researchers call for future hospitals to be designed and operated with a focus on using resources efficiently and minimizing the environmental impact of healthcare services [31]. For example, this can involve using sustainable building materials, implementing energy-efficient systems and technologies, and adopting environmentally friendly practices in areas such as waste management and transportation. Other areas of sustainability in healthcare include avoiding non-recyclable waste, especially plastics and disposable products, which play a particularly important role in sterile hospital environments [32]. For smart hospitals, sustainability must include digital systems and their often substantial energy demand [33]. Thus, smart hospitals also must become green hospitals [34, 35].

#### the Ecosystem Dimension of Smart Hospitals

Smart hospitals will increasingly extend their scope beyond their physical location to become integral parts – and potentially even the orchestrators – of broader healthcare ecosystems. Clearly, the healthcare system and its interaction with non-healthcare actors and stakeholders are evolving [3, 36], as new (digital) technologies and actors enter the scene. Smart hospital organizations must embrace this as an opportunity and nurture a broader ecosystem of stakeholders beyond their own organizational boundaries. These stakeholders include other healthcare providers, insurance companies, pharmaceutical and medical device companies, tech companies, and patient advocacy groups [37]. The importance of this ecosystem dimension is likely to increase in the age of AI, because data sharing is key to training AI systems. Decentralized and isolated hospital structures inherently lack necessary data, such as longitudinal datasets. Not only is interface openness and interoperability crucial, but also the involvement of all relevant stakeholders to pave the way for widespread AI acceptance and usage [24, 37]. This interplay can enable innovation, especially in technological and AI-driven environments [38], and can lead to a collective intelligence [39]. Multiple studies have shown that open ecosystems enable greater space for knowledge exchange, collaboration, and partnering. Therefore, open ecosystems foster innovation [2, 40–42]. Openness to ecosystem partners may also help smart hospitals become more resilient and better able to act in times of uncertainty [43]. As such, the future of smart hospitals is likely to be one embedded in broader ecosystems.

#### The human-centered dimension of smart hospitals

Finally, but perhaps most importantly, smart hospitals must be inherently human-centered, using digital technologies for the benefit of staff and patients. Human empowerment is seen by many as “*one of the core features of healthcare*” [44]. As the role of AI in hospitals grows, it also affects patients. For example, it can empower them by conferring greater data transparency and health literacy [45]. Researchers predict that AI will change not only the experience for all stakeholders involved, but also may shift professional roles and identities [46–49]. Therefore, researchers and practitioners alike expect a shift in job profiles and qualifications to accommodate technological advancements that augment or automate parts of the healthcare process [50].Completely new job profiles could emerge, for example, incorporating concepts such as home treatment. We see human-centeredness as an integral part of our conceptual framework of smart hospitals. Human-centeredness shapes how smart hospitals (1) employ co-creating tools and software with patients and other stakeholders [51, 52], (2) develop novel, often AI-augmented rather than fully automated treatment processes and services [53, 54] and more broadly (3) seek to improve patient experiences [55] and patient journeys [56].

To sum up, the concept and vision of smart hospitals rests on four pillars: AI usage, sustainability orientation, ecosystem embeddedness, and human-centeredness. However, neither practitioners nor researchers can predict to what extent – and when – this vision of smart hospitals might become reality. Knowledge about potential futures will enable today’s decision makers to shape tomorrow’s hospitals.

#### Prior forecasting studies

We identified three types of forecasting studies to guide decision makers interested in the possible futures of smart hospitals, though we found no single study forecasting the future of smart hospitals. We give a brief overview of these Delphi studies, identify important gaps, and show how our study can bridge these gaps, extending existing knowledge and creating a better understanding of the future of smart hospitals.

The first type of Delphi study focuses on a specific phenomenon, such as how AI helps identify individual diseases or treatment patterns, for example in the field of colonoscopy [57]. A second type of Delphi study focuses on the development of guidelines for the usage of AI in healthcare. Delphi studies have, especially related to clinical trials, provided multiple insights on how to improve the evaluation of AI systems [58–60], provide ethical use recommendations [61], or employ AI [62]. The third, and for this study most relevant, type of Delphi study focuses on the healthcare sector in general and its organizational and technological development trajectories. Table 1 provides an overview of the identified Delphi studies, with their corresponding relevance, scope, and limitations.

**Table 1:** Overview of relevant Delphi studies in healthcare focusing on tech and AI

AUTHOR & YEAR	STUDY TOPIC	SCOPE	KEY INSIGHTS	LIMITATIONS
Jungwirth & Haluza, 2019 [63]	Future of healthcare with information and communication technologies	73 experts from one country (Austria), 2 rounds	Importance of technologies in healthcare	Data collected in 2010 Broad scope
Liyanage et al., 2019 [64]	Design and evaluation of AI applications in healthcare	20 experts from 9 countries, 3 rounds	AI in healthcare needs to be evaluated, safe and effective	Delphi participants did not have much clinical experience with AI tools, limited and declining sample size (*n* = 20//12/8 experts in round 1/2/3)
Ravensbergen et al., 2019 [65]	eHealth as future trend in healthcare	16 experts from one country (Netherlands), 2 rounds	Preliminary digitalization tools for AI usage	Focus on elderly care, not maximal care, and limited sample size of clinical experts
Blease et al., 2020 [66]	Importance of machine learning for primary care	13 experts from 5 countries (focus USA), 3 rounds	Need for digital health and AI literacy for patients and physicians	Forecast period to 2029, limited sample size with 81% of participants from the USA
Ermolina & Tiberius, 2021 [67]	Impact of conversational AI onto staff	27 experts from multiple countries	Conversational AI may have an important role in hospitals	Forecast timeframe of 5 years, specific study scope targeting voice-enabled intelligent personal assistants
Lemmen et al., 2021 [22]	Systems medicine in healthcare	33 experts from one country (Germany), 2 rounds	Further research onto feasibility for systems medicine needed	Less focus on AI and the challenges hospitals will face, instead a strong focus on predicting developments in systems medicine in Germany up to 2030
Orkin et al., 2021 [48]	Task shifting and sharing as solution for healthcare worker shortage	15 experts from 6 countries, 3 rounds	Lack of inclusion of AI in established task shifting and sharing concepts	Delphi panelists were mainly recruited from university faculties or research institutes. Delphi study focuses on perspectives from low and middle income countries
Shinners et al., 2021 [49]	AI perception of healthcare staff	8 experts from 1 country, 3 rounds	User-centered approach of AI usage in healthcare needed	Limited sample size of 5 healthcare professionals and 3 IT experts and limited accuracy of predictions in Delphi study conducted
Deng et al., 2022 [68]	Smart health framework proposal	10 experts from one country (China), 3 rounds	Role of technology and AI in smart health	Limited sample size and focus on a region of China where a smart health model has been developed, focusing on the interplay between health actors, policy, technology, management and services
Lam et al., 2022 [46]	Digital surgery and use of new technologies	38 experts from multiple countries	AI indirectly plays an important role in the application of digital surgery	Clear focus on the evolution of surgery through digital technologies and the interplay between data, applications and analytics, rather than other healthcare related challenges
Koebe & Bohnet-Joschko 2023 [69]	Digital transformation scenario forecasting in the healthcare sector until 2032	58 experts (focus Germany), 2 rounds	Digital transformation of hospitals, focusing data usage, business models, regulation and technology	Moderate forecast timeframe to 2032 with focus on structures, regulations, business models and digital technologies, less on AI

Jointly, these studies highlight the increasing importance of technology and AI in healthcare. While these studies provide important snapshots of specific topic areas, the research literature still lacks a systematic and integrated analysis of the four dimensions of smart hospitals.

## Methods

Given the future-oriented perspective of our research questions, we rely on the Delphi method for forecasting and scenario development. Forecasting and scenario building are systematic and methodical approaches to preparing management decisions [70], strategies, and action plans [71]. Forecasting involves predicting future events or trends based on current data and analyses. Scenario development involves creating descriptions of potential future events or outcomes based on a variety of assumptions, opinions and variables [72]. Forecasting technological developments or changes can be particularly challenging for companies and managers. [73]. Scenarios should thus function as reliable visions of the future [74] and should include varieties of future scenarios in staged time horizons. It is especially important to keep in mind that a strategy is not a single roadmap that linearly guides organizations into the future. Instead, a strategy provides the means to create an attractive market position and operate in stable ways [75]. A Delphi study as an established forecasting method can help identify, construct, and clarify the future to a certain extent [76]. Developed by the RAND Corporation decades ago, the method uses repeated questionnaires and interviews to build expert consensus about future events [77]. Delphi studies involve multiple rounds of expert interviews to reach consensus. They can be conducted in a fully digital format, without face-to-face interviewing [78]. Such approaches allow experts to adjust and update their opinions in real time [79]. Given the ability of Delphi studies to provide expert consensus, enhance the accuracy of predictions through iterative feedback, and foster collaboration among diverse stakeholders, we decided to follow this approach for our study.

**Fig. 1:**
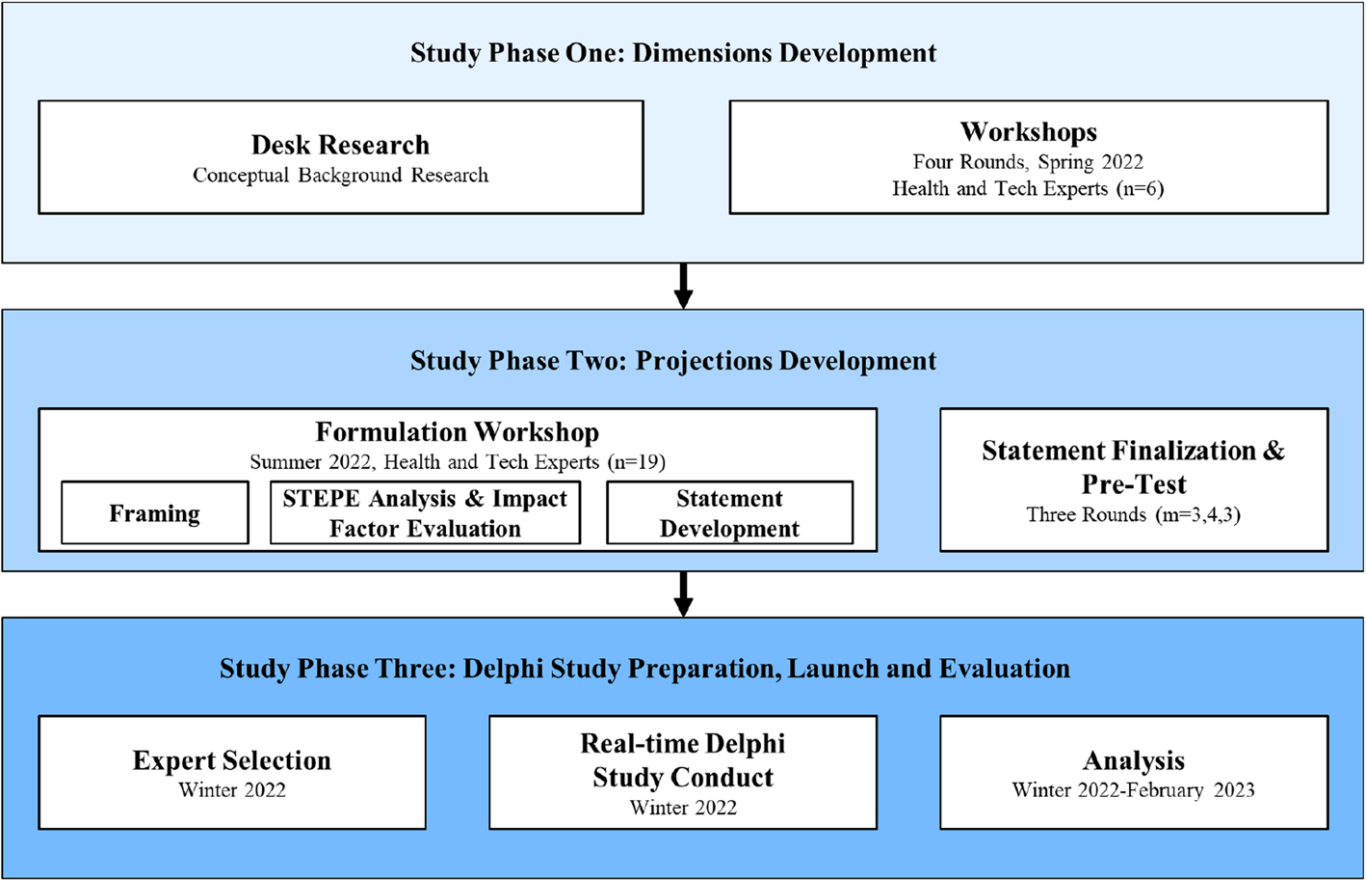
Smart hospital Delphi study design (adapted from [80–82])

Our study followed three main phases (see Fig. 1), [8, 83], which we describe in greater detail below. Initial data collection began in February 2022 and the three phases were completed in January 2023.

### *Phase one: validation o*f smart hospital dimensions

In phase one, we focused on validating and refining our conception of smart hospitals and its constitutive dimensions to obtain a robust baseline for our Delphi study. The extensive desk research, which contained a scoping review presented in the conceptual background, identified four important dimensions of the smart hospital concept. In four workshops (90–120 min long) with six experts, we sought to validate and refine this framework using six open-ended questions: 1) What defines a digital hospital? 2) What defines a future smart hospital? 3) What distinguishes a digital hospital from a smart hospital? 4) How will value be created in hospitals in the future? 5) How can hospitals become smart? 6) What statements can we derive that can measure current initiatives intended to develop smart hospitals?.

**Fig. 2:**
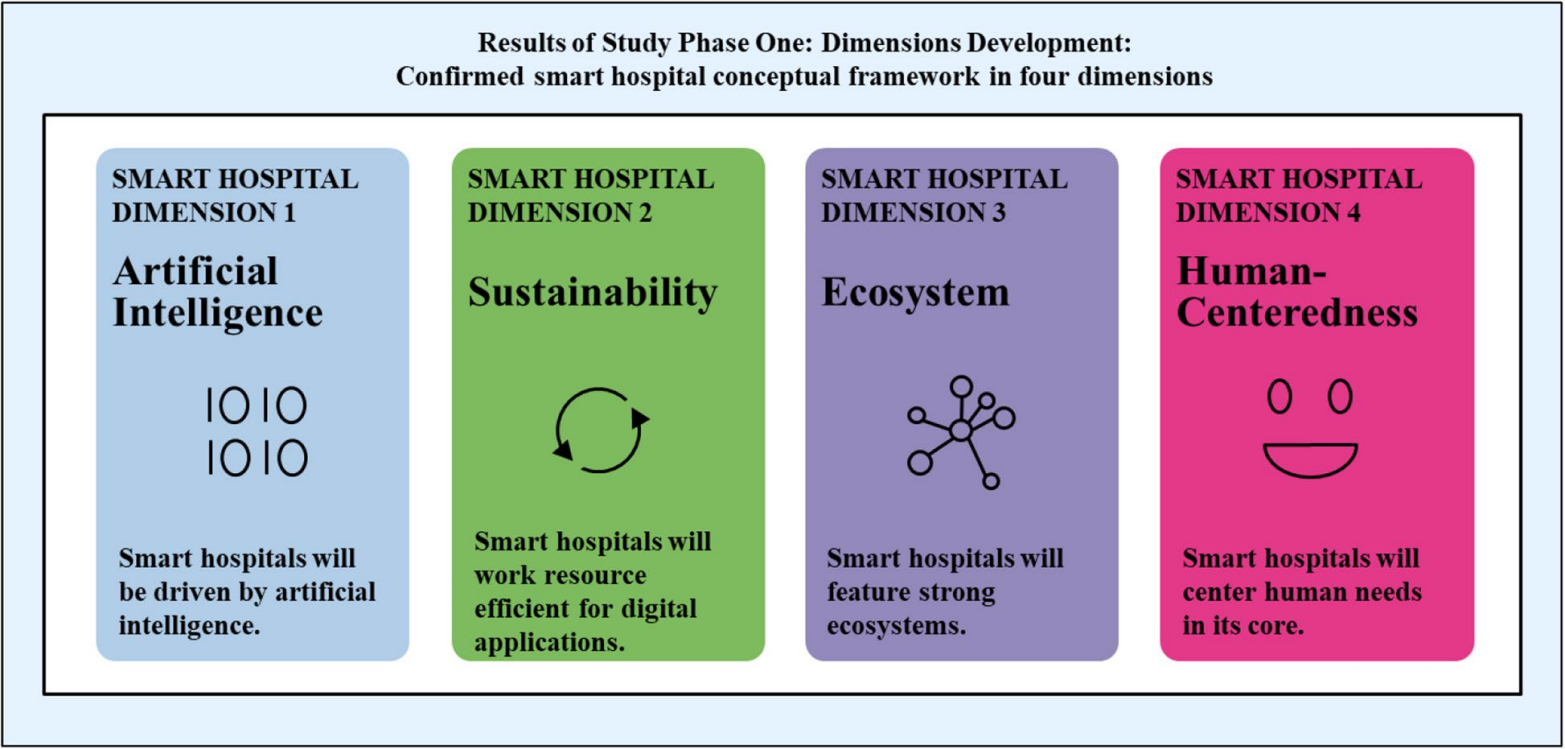
Results of study phase 1: dimensions development

Workshop participants discussed the characteristics and dimensions of the smart hospital from their perspectives. Overall, they confirmed the relevance of the four main dimensions: *artificial intelligence,* s*ustainability, ecosystems and human-centeredness*. We were able to map most of their statements and comments to one or more of the dimensions summarized in Fig. 2. Finally, we iterated back to literature, updating the conceptual background of our study based on the insights received during the workshops.

### Phase two: projections development

**Fig. 3:**
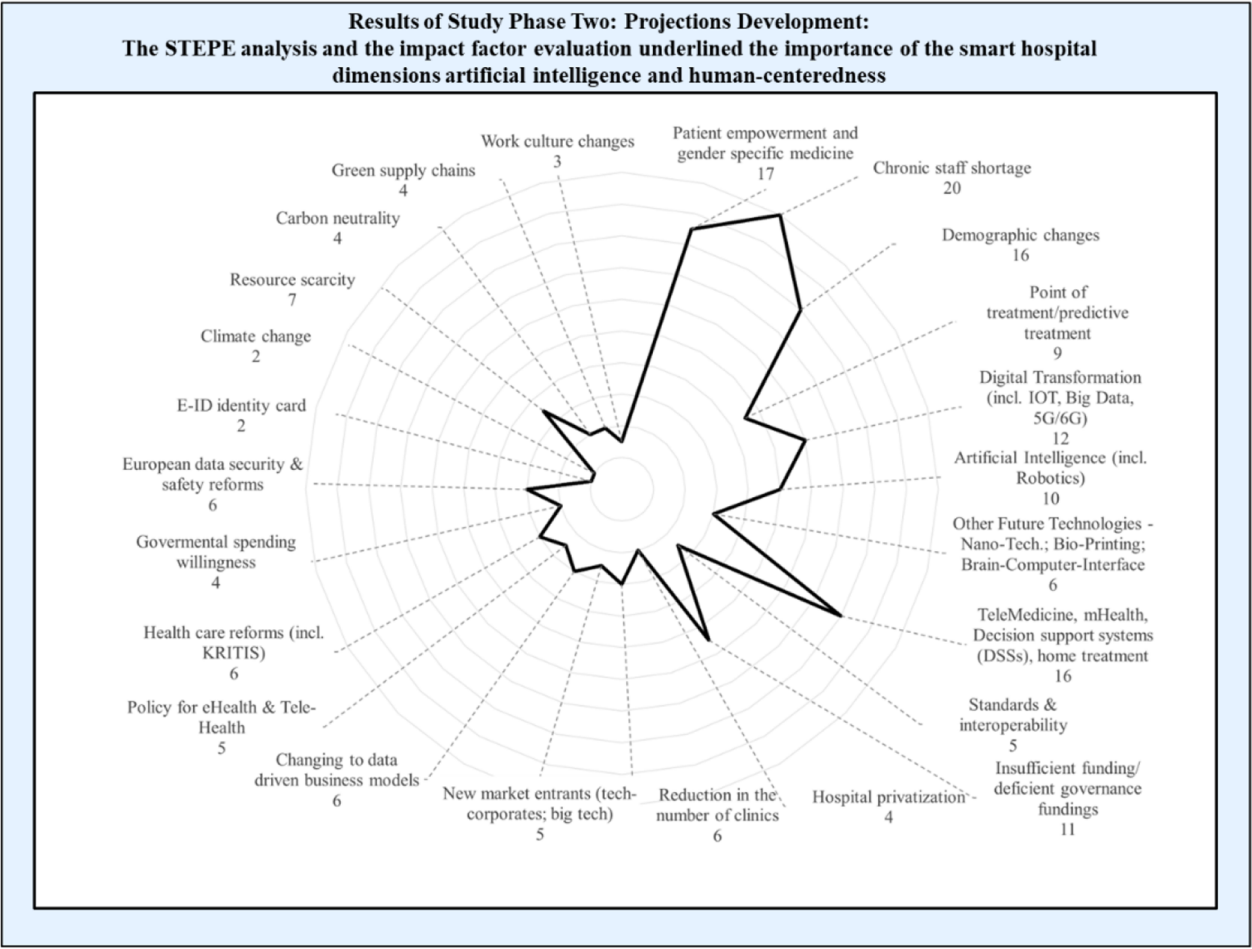
Results of study phase 2: STEPE analysis and impact factor evaluation

Based on the results of the first phase, we conducted a workshop with 19 healthcare and technology experts. We asked them to name factors that could affect the future of smart hospitals and then develop comprehensive projection statements within the four main dimensions. The workshop consisted of sequential steps, including a presentation of a STEPE framework to identify higher-level trends, a timeline of past innovations, and group discussions. The STEPE framework is frequently used for business analyses and has been helpful in developing Delphi projections [8, 83]. During the first step, we asked participants to identify technologies likely to shape the future of smart hospitals [84]. In the second step, we presented the STEPE-framework and gave experts time to add relevant sociocultural, technological, economic, political and ecological impact factors. During the following group discussion, we created a final set of impact factors. Each expert received 10 points and had to distribute them according to his or her individual assessment to evaluate the importance of the impact factors [85]. Summing up the points for each of the five factors separately resulted in the following ranking of their importance: sociocultural factors (34%/65 points); technological factors (26%/49 points), economic factors (19%/36 points), political and legal factors (12%/23 points) and ecological factors (9%/17 points) (see Fig. 3). The results show that the highest-ranked single STEPE factor is chronic staff shortage (20 of 190 points). The second highest factors are patient empowerment and gender-specific medicine (17 points), and last are demographic changes and telemedicine (16 points each). This finding is particularly interesting considering the importance that might be assigned to two of the four smart hospital dimensions: artificial intelligence and human-centeredness.

In the third step, we presented a futures cone model to the workshop participants. In the futures cone model [86, 87] potential futures are extrapolated in ranges of certainty from plausible to possible to wildcard ranges, including the important aspect of desirability [88]. We used the futures cone as a tool to guide the experts and their thinking in five-year steps from today to 2042.

The workshop participants then split into four groups of approximately the same size based on the participants’ expertise in the four main smart hospital dimensions (AI; human-centered; ecosystems and sustainability). Given background information, the four groups developed statements about how they envision the future of smart hospitals from 2027 to 2042 related to each dimension. Researchers and participants then discussed these statements and further refined them in a moderated group session. After the conclusion of the workshops, we processed the results and discussed them with an extended group of different experts in three rounds (*n* = 3;4;3). In the final development of the statement projections, we defined a total of 23 projection statements distributed across the four dimensions presented in Table 2.

**Table 2:** Final projection statement overview

	**#**	**PROJECTION STATEMENTS – FLOATING- AND FIXED-TIME HORIZON**	**PROJECTION DOMAIN**
**Dimension 1:** **Artificial Intelligence**	1	Artificial Intelligence (AI) use in relevant processes of administration, diagnosis, and treatment contributes to more efficient, quality-improved, patient-oriented and better planned activities in the hospital	Use of AI for treatment
	2	In-hospital (AI-assisted) prediction, diagnosis, and treatment of disease is performed with multimodal input data	Use of AI with multimodal data
	3	AI-supported sensors record important parameters of inpatients and provide early warnings	Sensory monitoring
	4	AI-based solutions that serve direct patient interaction (e.g., voice assistants in the patient's room) are primarily established as individual health services/ add-ons in hospitals	AI in patient interaction as paid services
	5	AI enables the application of systems medicine as part of personalized medicine. Hospitals use individualized medicine in standardized, scalable procedures to provide patients with precisely tailored and targeted treatments	Use of AI for systems medicine
**Dimension 2: Sustainability**	6	The use of AI enables an energetically self-sufficient and efficient use of energy in the hospital	Energy efficiency through AI
	7	Treatment and administrative processes (especially AI-based processes) are designed to conserve resources and are climate-neutral in terms of energy	Energy efficient process design
	8	Hospitals are energy resilient and (AI-based) systems are secure against crises and attacks	Crisis resilience
	9	^a^In 2027, energy price fluctuations and resource scarcity lead to a significant increase in (treatment) case costs and insurance premiums for patients	Impact of climate change on patients
**Dimension 3: Ecosystems**	10	Market participants are changing the market position and service structure of hospitals with new, including AI-based offerings and services	Changes in the structure of hospital services
	11	Hospitals are intersectorally connected via uniform standards; they collect, exchange and process data, also for the use of AI solutions	Intersectoral connection via standards
	12	Maximum care hospitals develop digital and AI-supported solutions in sustainable business models with partners of their own ecosystem	Collaborative business model development with partners
	13	Maximum care hospitals in Germany have an elaborated, written, communicated, lived and agile future strategy	Strategy & ecosystem
	14	^a^In 2032, treatments will increasingly take place in several treatment centers at different locations (in the hospital, with specialists and in medical centers)	Decentralized medicine in 2032
	15	^a^In 2042, the number of hospitals has steadily decreased, so that predominantly only maximum care hospitals and specialist or private hospitals remain	Near extinction of small hospitals in 2042
	16	Home treatment is an established part of the patient journey and hospital services	Home treatment is an established part of hospital services
**Dimension 4: Human-Centeredness**	17	Gender-specific medicine is well established in German hospitals. There is active work to resolve data bias and accumulate needed data to improve diagnoses and treatments	Gender specific medicine
	18	Patients act as facilitators of their own care and treatment process through existing data & health literacy	Data & health literacy of patients
	19	^a^In 2032, direct doctor-patient communication will no longer be necessary for preliminary discussions and standard diagnoses; any clarification will be provided by trained specialist staff	Change in activity profiles through the upgrading of tasks in 2032
	20	^a^In 2037, new and changed roles and professions have entered hospitals: Due to new technologies (especially AI) and the increasing complexity of treatments and diseases, the task and activity profiles have become increasingly granular and specialized	Development of new job professions and roles in 2037
	21	^a^In 2032, the number of general practitioners in private practice has decreased significantly, which leads to a deterioration in rural care	General practice shortage in 2032
	22	^a^ In 2027, growing staff shortages and rising case numbers will lead to even longer waiting times and inadequate care in hospitals	Growing staff shortage and workload increase in 2027
	23	^a^In 2032, treatment cases are predominantly geriatric, chronic and multimorbid in nature, and at the same time the burden on hospital staff and processes reaches a peak	Capacity overload due to aging & multimorbidity in 2032

Statements that are marked with (^a^) have been measured in fixed-time horizon

Notably, some statements were associated with multiple dimensions. We opted to assign such statements to their dominant theme and the corresponding primary dimension of the smart hospital. We intentionally excluded other topics and dimensions, including non-AI-related technology areas. Among the final 23 projection statements, we formulated 15 as floating-time-horizon statements and eight as fixed-time-horizon statements [80]. To choose either a fixed or floating horizon, we conducted discussions during the finalization and pre-test workshop and considered expert estimations regarding the uncertainty of the occurrence of each projection statement. If a consensus regarding the anticipated occurrence in one of the years 2027, 2032, 2037, or 2042 emerged during initial workshop discussions, we adopted a fixed-time horizon approach with the corresponding estimated year.

### Phase three: preparation, launch, and evaluation of the real-time Delphi study

In the third phase, we prepared and launched the real-time Delphi study based on the 23 projection statements. Healthcare Delphi studies typically include two to three rounds of statement discussions and consensus-seeking [89]. However, it is not unusual for Delphi studies to work with a real-time approach and a mixture of statement types regarding their time-horizon [80]. We chose the real-time approach because we assumed that participants would have limited time to complete the study and revise changes. With a round-based approach, we saw the potential issue that participants would drop out of the study if consensus had not been reached after one or two rounds. With the real-time approach, participants were able to access the study at any time, see new progress and adjust their own answers based on new comments. We sent multiple reminders with different focus areas of the study to address under-reported parts. We chose Calibrums Delphi-software Survelet, which has been the object of analysis in the field, and has proven useful for our purposes [78, 80, 90].

#### Expert selection for the real-time Delphi study

For this study, the expert selection process involved identifying, selecting, and contacting relevant experts in the healthcare sector. We used the criteria of being interested in forecasting future developments in hospital transformation and having knowledge in the fields of healthcare and AI, innovation, or technology management. We contacted experts directly as well as indirectly through healthcare clusters and networks in the German-speaking region. While striving to maintain high expertise among participants [91], we anticipated a low response rate for experts working in the clinical field due to the high workload. For this reason we used the snowball sampling method [92]. A total of three subclusters emerged as heterogeneous expert groups [90] with a minimum target size of *n* = 10. The three groups included 1) hospital employees in decision-making positions for new technologies and transformation processes, 2) employees in science and science-related fields with expertise in AI and health, as well as founders and general managers in the health tech sector. We sent a total of 300 personal invitations with a total response of 56 (18.67%). Of those who responded, 42 experts (14%) started the study and a total of *n* = 39 completed the study with a distribution of 30.8% female, 64.1% male and additional 5.1% non-binary experts (see Table 3).

**Table 3:** Demographics of Delphi study participants

DEMOGRAPHICS OF DELPHI STUDY PARTICIPANTS (*n* = 39)	
Panel • 300 Invitations sent • 56 Initial invitation replies • 42 Experts started the study • **39 Experts completed the study**	Size of work organization • **35.9% > 10,000 employees** • 12.8% 5,001 – 10,000 employees • 12.8% 1001 – 5,000 employees • 15.8% 201 – 1,000 employees • 17.9% < 200 employees
Highest education • **53.8% PhD / MD** • 43.6% Master’s degree or higher diploma • 2.6% High school graduate	Field of work • **48.8% Science or research related** • 28.2% Health & technology provider /start-ups • 23% Hospitals / directly in health care
Gender • **64.1% Male** • 30.8% Female • 5.1% Non binary	Professional experience • 16.6 years on average
Current job position • **35.9% Executives, head physicians** • 30.7% Employees in private companies (health or tech related) • 23.1% Scientists / Researchers • 10.3% Professors	Reported knowledge areas of the experts (multiple entries possible) • **53.9% in artificial intelligence** • 51.3% in digitalization • 46.2% in medicine; 28.2% in biomedicine • 43.6% in sustainability • 41% management of organizations • 30.7% in social science

These 39, all German-speaking experts, have an average of 16.6 years of professional experience. At the time of the study, 35.9% are working as CEOs, general or technical managers, or chief physicians; 23.1% as scientists, 10.3% as professors and 30.7% in project teams in private or semi-private companies. 23% of experts are working in hospitals or care facilities, 28.2% in technology- and health organizations and startups, and 48.8% in research (near) institutions. As their highest educational level, 53.8% of all experts have a doctoral degree, 43.6% a master’s degree and one expert (2.6%) has a high school degree. 46.2% of experts declared basic up to expert knowledge in the field of medicine and 28.2% in biomedicine, 51.3% in the field of digitalization and 53.9% in the field of artificial intelligence. This was followed by 41% of experts indicating confidence and high knowledge in management, 43.6% in sustainability and 30.7% in social science. Therefore, the expert pool represents a variety of knowledge fields relevant to forecasting the future of smart hospitals. 48.7% of all experts are working in organizations with more than 5000 employees, followed by 33.4% in organizations with between 200 to 5000 employees, and 17.9% in organizations with < 200 employees.

#### Measurement of statements and evaluation of the Delphi study

We used two distinct approaches to measure and evaluate the Delphi study projections. For the first group of fixed-time horizon statements (see Table 2—#9; #14; #15; #19; #20; #21; #22; #23) the time period was set in the projection statement. Thus, we measured the probability of occurrence with a 5-point Likert scale (1—very low to 5—very high) along with the expected impact on hospital management and strategy and desirability of occurrence. For fixed-time horizon statements, we define a consensus as reached if the estimated occurrence mean is at least 80% with an interquartile range (IQR) of < 1.25. We used the same approach to define a consensus for impact and desirability (mean > 80%; IQR < 1.25). Statements that reached consensus with mode ≥ 50% and IQR < 1.25 for occurrence, impact or desirability have the strongest consensus indication. For each statement, experts were excluded if they did not report each occurrence, impact, and desirability. For the floating-time horizon statements (see Table 2—#1–8; #10–13; #16–18), it was necessary to measure them according to the expected year of occurrence, as the time period was not pre-defined in the projection statement. Therefore, measurement was performed according to the expected year of occurrence on a 5-point-likert scale (1- in 5 years /2027 to 5 – in 25 years/after 2042) and expected impact and desirability of occurrence equally using 5-point-likert scales. Our measurement of occurrence, impact, and desirability of occurrence was guided by relevant literature and existing Delphi studies [8, 93, 94]. We defined a consensus for floating-time horizon statements for estimated occurrence with a mode of at least 50% of answers. Appendix 1 includes the methodology of the bias analysis and the analyses of dissent and qualitative comments.

#### Cluster analysis

As a last step, we analyzed the Delphi results to identify potential clusters [94]. These clusters show consistent answers throughout the expert judgments, thereby indicating potential consistent scenarios. Appendix 1 gives further technical information about the cluster analysis.

### Results for Delphi projections

**Fig. 4:**
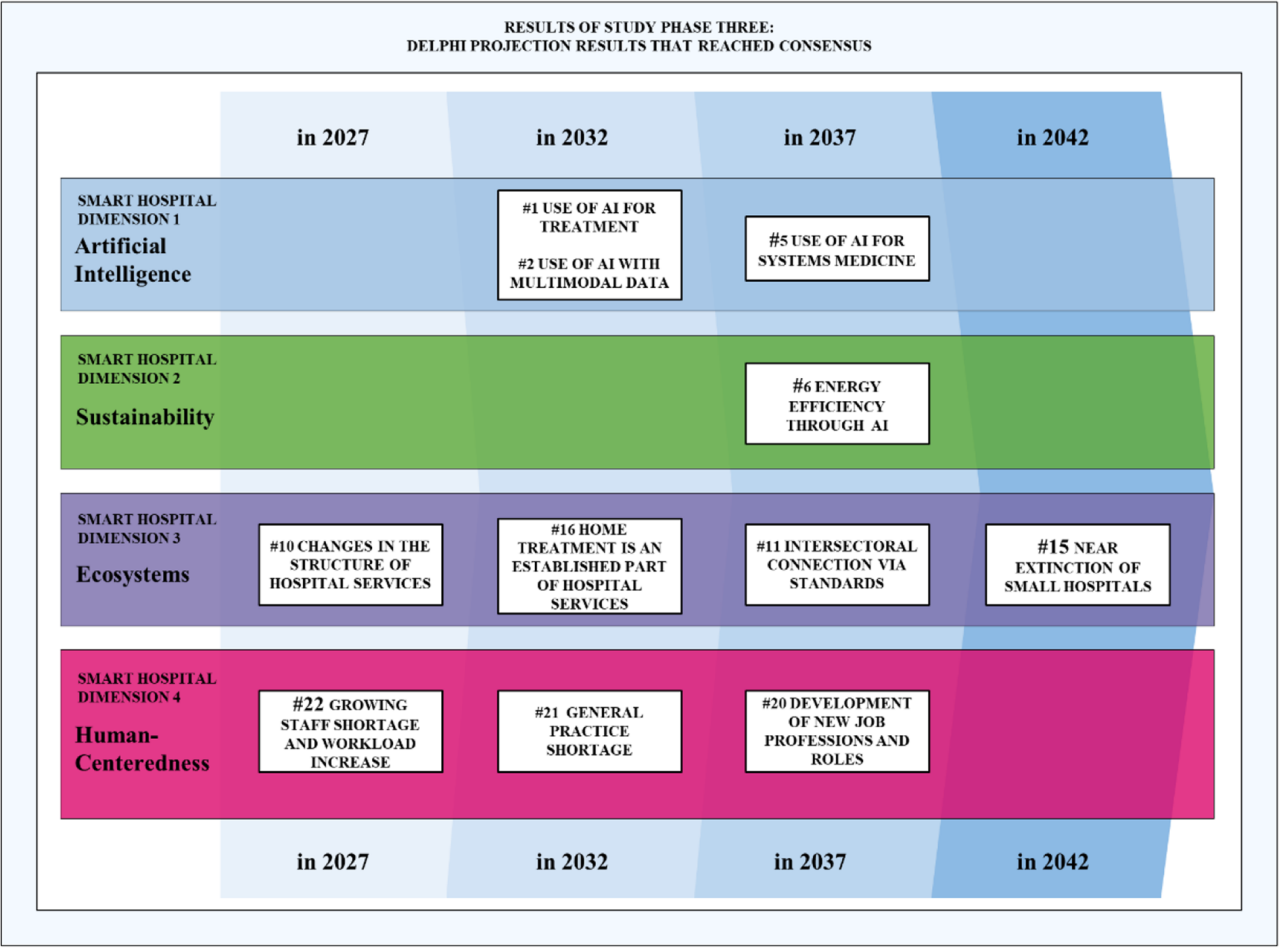
Visualization of Delphi projection results that reached consensus

All 39 experts discussed the 23 projections and reached a consensus for a total of 11 projections across all four dimensions. Figure 4 shows an overview of the final 11 projections, positioned as a function of their dimension and time horizon, and providing a multi-dimensional and future-oriented view of the projected evolutionary path of smart hospitals.

We identified a consensus for seven floating-time horizon statements (#1; #2; #5; #6; #10; #11; #16), where the mode frequency is at least 50% of all responses in that projection. For statements with a fixed time horizon (#15; #20; #21; #22), more than 80% of respondents agreed that they were likely to occur within the given time frame. Table 4 gives an overview of the results.

**Table 4:** Statistical results overview

#	PROJECTION DOMAIN	n	EP (MODE)	MODE FREQ	EPT (MEAN)	IQR	(I)	IQR	(D)	IQR
**Dimension 1: Artificial Intelligence**										
**1**	**Use of AI for treatment**	**35**	**2032**	**61.1%**			**3.77**	**1** ^a^	**4.23**	**1** ^a^
**2**	**Use of AI with multimodal data**	**35**	**2032**	**66.7%**			**3.89**	**0** ^a^	**4.51**	**1** ^a^
3	Sensory monitoring	35	2027	40%			3.40	1	4.23	1
4	Use of AI in patient interaction as paid services	29	2032	32.3%			2.52	1^a^	3.03	2
**5**	**Use of AI for systems medicine**	**28**	**2037**	**56.7%**			**3.75**	**2**	**4.14**	**1**
**Dimension 2: Sustainability**										
**6**	**Energy efficiency through AI**	**26**	**2037**	**53.3%**			**2.92**	**1**	**4.08**	**2**
7	Energy efficient process design	26	2037	37.9%			3.23	1	4.5	1
8	Crisis resilience and robustness	26	n/a	30.8%			3.42	1	4.54	1^a^
9	Impact of climate change on patients in 2027^b^	33			71% 3.55	1	3.88	2	1.42	0^a^
**Dimension 3: Ecosystems**										
**10**	**Changes in the structure of hospital services**	**29**	**2027**	**53.3%**			**3.55**	**1**	**3.72**	**1**
**11**	**Intersectoral connection via standards**	**30**	**2037**	**50%**			**4.28**	**1**	**4.62**	**1** ^a^
12	Collaborative business model development with partners	29	2032 -2037	32.3%			3.24	1	3.86	1
13	Strategy & ecosystem	29	2037	33.3%			3.62	1	4.21	1
14	Decentralized medicine in 2032^b^	31			67% 3.35	1	3.58	1	3.58	1
**15**	**Near extinction of small hospitals in 2042** ^b^	**32**			**81%** **4.06**	**1**	**4.19**	**1**	**2.78**	**3**
**16**	**Home treatment as is an established part of hospital services**	**29**	**2032**	**66.7%**			**3.72**	**1** ^a^	**4.28**	**1** ^a^
**Dimension 4: Human-Centeredness**										
17	Gender specific medicine	29	2032 -2037	34.5%			2.86	1	4.31	1
18	Data & health literacy of patients	29	2032	26.7%			3.31	1	4.17	2
19	Change in activity profiles through the upgrading of tasks in 2032^b^	33			64.2% 3.21	1	3.33	1	3.39	1
**20**	**Development of new job professions and roles in 2037** ^b^	**33**			**82.4%** **4.12**	**1**	**3.97**	**2**	**3.67**	**1**
**21**	**General practice shortage in 2032** ^b^	**33**			**80%** **4.0**	**2**	**3.91**	**1** ^a^	**1.58**	**2**
**22**	**Growing staff shortage and workload increase in 2027** ^b^	**33**			**86.6%** **4.33**	**1**	**4.42**	**1** ^a^	**1.33**	**0** ^a^
23	Capacity overload due to aging & multimorbidity in 2032^b^	33			72.2% 3.61	1	4.0	2	1.91	2

Delphi projection results marked in bold reached consensus

*n* number of participants, *EP* estimated probability of occurrence, *EPT* estimated probability time of occurrence

n for all (Y;I;D); Mode ≥ 50% = consensus; Mean ≥ 80% = consensus for expected probability in 1–5 (nr) and showed as % for mean as occurrence probability derived from 5-point-likert scale; (^a^) if IQR < 1.25 and if Mode ≥ 50%. (I) = expected impact. (D) = desirability. Statements that are marked with (^b^) have been measured in fixed-time horizon

### The artificial intelligence dimension of smart hospitals

Our Delphi results suggest that hospitals are likely to make notable progress with regards to the AI dimension of smart hospitals by 2032. More specifically, there is a consensus that smart hospitals will leverage AI to augment key administrative, diagnostic, and treatment processes in a way that improves efficiency, quality and patient-centricity (#1) by 2032. Experts also concur that, by 2032, smart hospitals will utilize multimodal input data for AI-assisted prediction, diagnosis, and treatment of diseases (#2). Such advances will go beyond today’s limited AI applications and unlock substantial clinical value. Given the speed of progress in AI, experts also anticipate smart hospitals to use AI for delivering personalized precision medicine and treatment tailored to the characteristics of each patient, by 2037 (#5).

### The sustainability dimension of smart hospitals

Expert assessments are more heterogeneous regarding smart hospitals’ future sustainability. Indeed, only one of the four projection statements achieved consensus among the experts. Notably, by 2037, smart hospitals are expected to use AI to enable more efficient energy use in hospitals (#6). Eventually, AI use in smart hospitals might change from a substantial net consumer of energy to a meaningful enabler of energy efficiency, highlighting that the visions of smart and green hospitals might eventually converge.

### The ecosystem dimension of smart hospitals

Most experts agree that smart hospitals will play a central role within their broader healthcare ecosystems. As smaller hospitals continue to face near extinction by 2042, the smart hospitals of the future will tend to be maximum care facilities or specialty clinics with considerable influence in their ecosystem (#15). By 2032, smart hospitals will have integrated home treatment as part of their standard services. This illustrates that smart hospitals will extend beyond their boundaries into local communities (#16), a projection seen as both impactful and desirable. Likewise, new players such as start-ups will enter these ecosystems and infuse them with new digital solutions that enable smart hospitals to operate more effectively and/or efficiently (#10). However, common standards, intersectoral connectivity, and interoperability as prerequisites for processing and sharing data are not expected to be realized before 2037. This illustrates the scope of the challenge (#11).

### The human-centered dimension of smart hospitals

Smart hospitals of the future will leverage digital technologies to redefine structures, processes, and roles to put employees and patients center stage. In doing so, smart hospitals will have defined new professions and roles by 2037 that enable high-quality, patient-centered care at scale (#20). Staff shortages will increase and hospital workloads will continue to grow until 2027 (#22), as along with a general practice shortage until 2032 (#21). However, other projections associated with the human-centered dimension of smart hospitals did not show a consensus among experts. These are, for instance, related to doctor-patient communication, gender-specific medicine, or patients as facilitators of their own care.

### Results from cluster analyses and scenario development

**Fig. 5:**
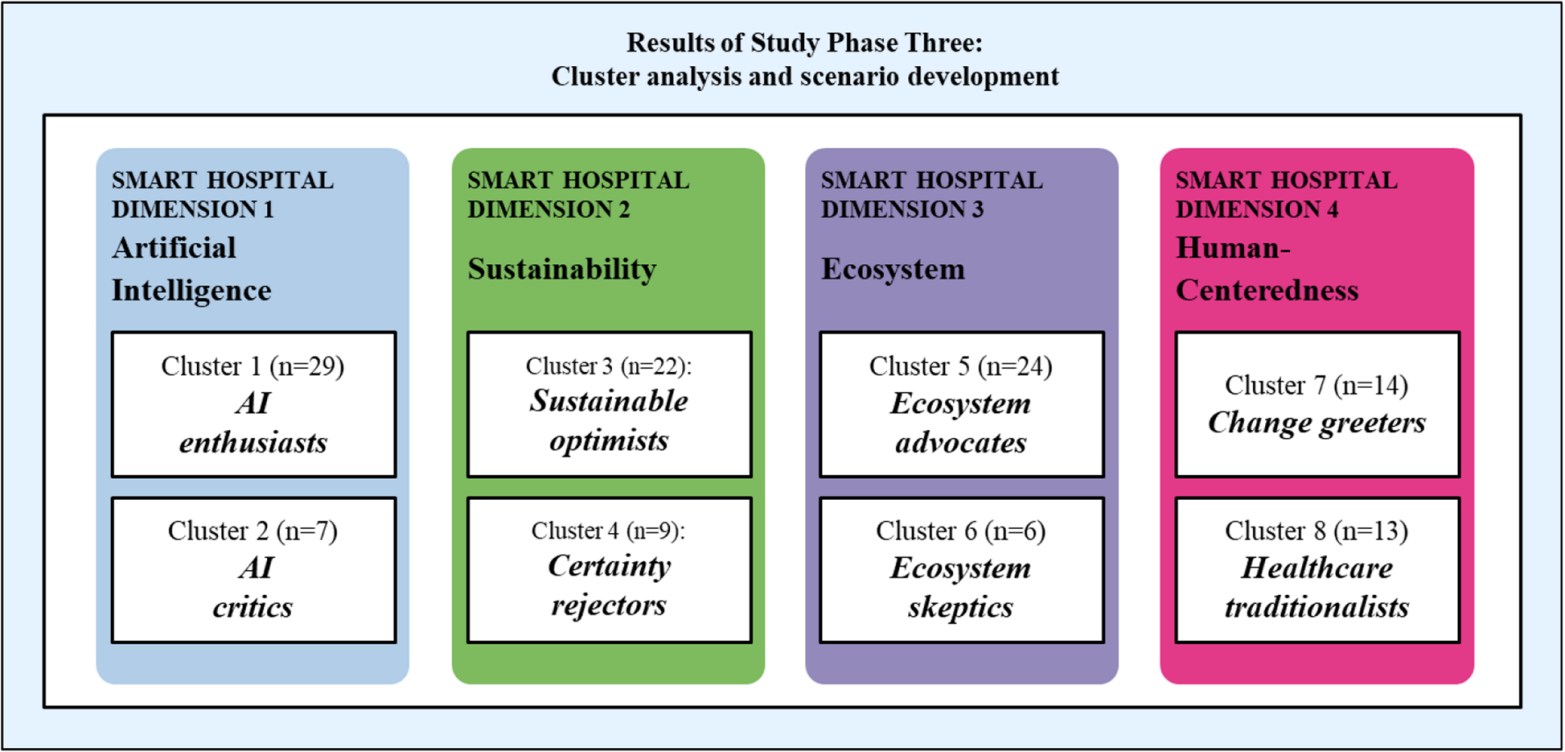
Cluster analysis results overview

Our cluster analyses identified two clusters for each of the four dimensions of the smart hospital summarized in Fig. 5 and further explicated below. Table 5 in the Appendix shows additional details regarding the statistical estimates of the cluster analysis.

### Artificial intelligence dimension: AI enthusiasts versus AI critics

As for the AI dimension of smart hospitals, experts in cluster 1 (AI enthusiasts, *n* = 29) expect smart hospitals to adopt AI earlier and with more impactthan experts in cluster 2 (AI critics, n = 7). Among AI enthusiasts, some experts suggested that AI-based patient interaction could be funded through reimbursement instead of paid for as a service (projection #4). AI enthusiasts anticipate the widespread adoption of AI-enabled systems medicine by 2037. No shared prediction emerged among AI critics.

#### Sustainability dimension: sustainability optimists versus certainty rejectors

Sustainability optimists (cluster 3, *n* = 22) see AI as key to improving energy efficiency (#6) and optimizing hospital processes. They predict full implementation by 2037. Sustainability optimists acknowledge high investment requirements but consider AI-driven energy improvements crucial for reducing consumption. Some, however, doubt that full energy self-sufficiency will be achieved. Despite these challenges, they view these advancements as highly desirable for the future of smart hospitals. In contrast, certainty rejectors (cluster 4, *n* = 9) express skepticism about AI’s role in energy efficiency (#6) and accordingly avoid firm predictions. Both groups express concerns about the cyber security of smart hospitals (#8). They wonder if ongoing vulnerabilities could prevent fully secure systems from being achievable. One expert referred to a “contest between system backups and attacks” (comment by expert #13) that may persist for the foreseeable future.

#### Ecosystem dimension: ecosystem advocates versus ecosystem sceptics

Ecosystem advocates (cluster 5, *n* = 24) anticipate smart hospitals to become key actors within their healthcare ecosystems by 2037. This will fuel new service development by 2027 (#10), joint business model development with ecosystem partners by 2032 (#12), and the setting of uniform standards for collecting, processing, and exchanging data within the broader ecosystem by 2037 (#13). In contrast, ecosystem skeptics (cluster 6, *n* = 6) are more doubtful about the necessity of ecosystems and whether the required development of common standards and strategies will occur by 2042. Regarding market shifts and new entries (#10), ecosystem advocates note these changes are visible today. Skeptics, however, expect them closer to 2032. Both groups agree on the high impact and desirability of intersectoral connectivity standards among hospitals (#11). Ecosystem advocates believe this process has already begun, citing slow progress due to past failures and lacking agility. In contrast, skeptics offer a bimodal view, with some expecting adoption by 2037 and others predicting delays until 2042.

#### Human-centered dimension: change greeters versus healthcare traditionalists

Change greeters (cluster 7, *n* = 14) view future developments in smart hospitals that could result in fewer doctor-patient interactions (#19) positively. Examples of such developments include increasing data and health literacy of patients (#18) and changes to healthcare workers’ activity profiles resulting from the upgrading of tasks. In contrast, healthcare traditionalists (cluster 8, *n* = 13) see these changes as less desirable. Change greeters predict gender-specific medicine (#17) to emerge around 2037, while traditionalists predict it by 2032, though both clusters exhibit uncertainty on the topic. Patient data and health literacy (#18) also reveal contrasting views. Change greeters expect this issue to arise earlier, noting ongoing efforts while acknowledging significant obstacles. Traditionalists predict a later development, around 2042, expressing skepticism about patients’ interest in data literacy.

## Discussion

This study illuminates a multidimensional concept of smart hospitals, with new insights into AI’s role and impact on future healthcare developments. Over 18 months, 39 experts from various disciplines delivered valuable insights for both research and practice. We began our analysis by identifying four relevant areas within the smart hospital literature to serve as a cognitive framework for our workshops. We used an iterative approach and concluded that all four dimensions—artificial intelligence (AI), sustainability, ecosystems, and human-centeredness—are essential to understanding a technology-driven smart hospital. With our second research question, we identified key challenges facing the German healthcare sector in the coming decades, exploring how AI can be further integrated into smart hospitals. AI is expected to play an increasingly critical role across various functions, including administration, diagnosis, treatment, and systems medicine. In these ways, AI will enable more personalized healthcare. Experts anticipate that AI’s capacity to process extensive multimodal data will transform hospital operations and facilitate personalized treatments by 2037. In terms of sustainability, experts see AI as pivotal for improving energy efficiency and optimizing hospital processes. However, concerns about whether full energy self-sufficiency can be achieved indicate a need for more research on AI’s role in the transition to sustainable practices in smart hospitals.

In terms of the ecosystems dimension, advocates foresee smart hospitals becoming integral to their healthcare ecosystems by 2037. They predict that new service developments will emerge by 2027 and business models will be developed jointly with ecosystem partners by 2032. In contrast, skeptics express doubt about whether ecosystems are necessary and whether common standards will arise by 2042.

In regard to the human-centered dimension, “change greeters” view developments such as decentralized medicine positively. However, “traditionalists” express concern about reduced doctor-patient interactions. This dichotomy highlights the need for consideration of how digital technologies may affect these relationships.

Our findings also indicate an increased workload alongside a decreased availability of staff by 2027. By 2032, hospitals may face capacity overload due to rising numbers of patients and ongoing shortages of physicians, a challenge evident today. Home care is expected to become integral to patient journeys by this time. Despite these advancements, our participants lacked consensus on issues such as gender data bias and how to empower patients through enhanced health literacy—areas identified as under-researched.

### Theoretical contributions

This study builds on a substantial body of literature on the potential of AI to shape future smart hospitals [28–30]. By identifying and analyzing four key dimensions—artificial intelligence, sustainability, ecosystems, and human-centeredness—we contribute a multidimensional framework that enhances our understanding of how these elements interact in the context of smart hospitals. This approach aligns with prior research emphasizing a holistic view of smart hospital development [24]. Previous Delphi studies have focused on specific AI applications or short-term outcomes [41] We address these limitations by researching challenges with AI integration across multiple dimensions relevant to smart hospitals. For instance, we show the contrasting perspectives between AI enthusiasts and critics regarding the adoption and impact of AI technologies. We also explain differing expectations of sustainability initiatives between optimists and rejectors. Our findings emphasize AI’s roles as a treatment technology and an integral component in most areas related to smart hospitals. This perspective resonates with discussions in the literature about technological advancements enhancing operational efficiency while maintaining patient-centric approaches in smart hospital frameworks [72]. Additionally, we identify under-researched areas critical for ensuring equitable access to services provided by smart hospitals. Gender data bias and health literacy are examples of such. We must address these gaps to foster an inclusive environment that empowers patients to engage in care processes. Our comprehensive analysis contributes to future-oriented research on smart hospitals by providing a conceptual framework to enrich previous findings on their transformation [69–72]. With this study, we have built a foundation for further research on integrating emerging technologies into smart hospital systems, while addressing ethical considerations and implementation challenges.

### Practical implications

Our findings anticipate increased specialization for smart hospital staff, driven by technological advancements and the integration of systems medicine by 2032. Early intervention to mitigate staff shortages or care imbalances due to emerging technologies will be crucial. Hospitals must adapt to changes in their service structures resulting from new market entrants. Understanding the implications of AI adoption in hospital settings is vital. This is especially true for the many institutions currently facing low maturity levels regarding technology integration [32]. Our Delphi study expert panel envisions a future for hospitals supported by AI and enriched with multimodal data. In particular, they expect home-based and individualized care to evolve alongside the rise of systems medicine. However, it remains unclear whether these opposing effects will counterbalance each other or which trajectory—positive or negative—will prevail. We encourage hospital managers and decision-makers to evaluate the advantages and disadvantages of these developments, taking into account when widespread adoption of systems medicine and home treatment can be anticipated and what resources will be required. Our findings highlight a need to examine patients’ roles in shaping their treatment pathways, contrasting with research that emphasizes staff perspectives. Future studies should focus on enhancing patient engagement while directly incorporating patient viewpoints into AI development within hospitals.

### Limitations and further research

With our Delphi study, we aimed to cover a broad range of dimensions related to smart hospitals. However, some projection statements proved ambiguous due to this thematic scope. Although we included all four dimensions—artificial intelligence, sustainability, ecosystems, and human-centeredness—this breadth led to projections applicable to multiple areas. Future studies could benefit from a more focused approach. Researchers could explore specific aspects such as nanotechnology or AI-enabled robotics in greater detail. Additionally, ethical considerations surrounding AI deployment in smart hospitals require further exploration [39]. Effective implementation would depend on issues such as data privacy and bias prevention. Researchers should assess how regulatory frameworks can be integrated into smart hospital operations while ensuring compliance with ethical standards. Our focus on the German context also limits the generalizability of findings. Each country may face unique challenges in transforming its healthcare system [69]. Therefore, more in-depth studies of the proposed dimensions of smart hospitals would help identify context-specific barriers. Comparative analyses with countries like the United States [17] and Israel [18], which have made significant advancements in healthcare technology, could yield useful insights for Germany. Investigating how diverse facilities evolve into smart hospitals with varying degrees of sophistication would also be beneficial. Ultimately, these inquiries could address key questions: Are maximum care providers the only institutions capable of becoming smart hospitals? How will AI reshape operational models? Such comparative case studies would enhance understanding of global trends in smart hospital development while developing best practices tailored to local needs.

## Conclusion

This study offers fresh perspectives on integrating AI, sustainability, ecosystems, and human-centeredness into the concept of smart hospitals in Germany. While we cannot definitively predict what these facilities will look like in 2042, our findings provide a framework for understanding AI’s potential for the coming decades. We highlight the challenges and opportunities that come with integrating new technologies while undergoing staffing shortages along the smart hospital transformation process. Finally, we urge stakeholders to critically assess the multifaceted implications of utilizing AI in the development of smart hospitals.

## Supplementary Information

Supplementary Material 1

## Figures and Tables

**Fig. 6:**
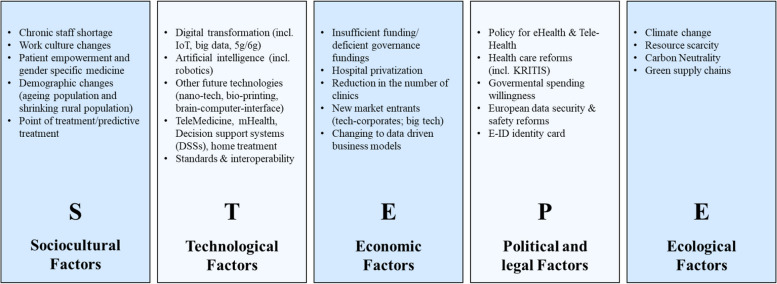
STEPE analysis for impact factors onto hospitals of the future

## Data Availability

The datasets generated and analysed during the current study are available from the corresponding author on reasonable request.

## Appendix 1

### Methodological Appendix

#### Bias analysis

One potential bias within Delphi studies can result if experts tend to estimate desirable futures as more likely or sooner to occur, whereas undesirable futures are estimated as more unrealistic or as likely to occur farther in the future [88]. To check for this potential bias, we performed a correlation analysis (bivariate – Pearson correlation coefficient, one sided) between estimated occurrence and desirability (occurrence here 1 – 5; years 2027-2042; and desirability 1 low – 5 very high). A high correlation indicates that an estimated early occurrence is more likely to be subjectively desirable. We only report correlations that are significant (sig <0.5 = *; sig <0.05 = **) and have a Pearson correlation > 0.49.

#### Dissent and qualitative comment analysis

Although Delphi studies aim to reach consensus, areas of dissent often emerge. It has been shown to be valuable to focus on dissent to look at conflicting outcomes and visions for the future [80, 94]. Therefore, we conducted an examination of dissent and qualitative comments.

#### Cluster analysis

We tested the data for the possibility of primary clusters over all 23 projections. We could not identify overall clusters that share an umbrella over all dimensions. We then checked for clusters in each of the four Delphi study dimensions separately. For this, we performed a two-step clustering approach using the software SPSS to identify the number of sub-clusters first, then applied k-means clustering analysis. We retested the identified cluster data groups in terms of standard deviation. Some experts were excluded from the cluster analysis due to too many missing values. For the sustainability, ecosystems and human-centered clusters, n=5,6,9 experts were excluded from the analysis pool, mainly due to lack of answers or discrepancies in the conduction of the cluster analysis. Overall, the cluster analysis identified two cluster groups for each of the four dimensions, revealing a total of eight clusters.

## Appendix 2

### Results Appendix

#### Results of the bias analysis

Within the bias analysis, we found significant correlation between occurrence and desirability for five statements (#5 (.551**); #7 (.502**); #11 (.616**); #17 (.566**); #18 (.502**)). However, of these five statements only #5, which focuses on systems medicine as a future field of *smart hospitals,* reached a clear consensus in the analysis. For this statement, it might be possible that experts tend to estimate the expected probability of occurrence higher because of high subjective desirability. With a mode frequency of 56.7%, we decided to keep statement #5 in the further analysis, but indicate that there might be a bias that reached consensus.

#### Dissent and qualitative comment analysis

We considered a total of 13 projection statements for the dissent analysis (#3; #4; #7; #8; #9; #12; #13; #14; #15; #17; #18; #19; #23), where predominantly no consensus was reached. Looking at the standard normal distributions of the frequencies of the experts’ statements, we observed dichotomous or trimodal distributions. We examined these distributions more closely in the next step by comparing the different group statements and analyzing the comments.

For projection #8 (crisis resilience and robustness), 18 experts did not provide an answer or excluded the answering, which leaves only 18 expert opinions for this statement (n=36 experts in total). Due to this, a dichotomous distribution (18 non-answers versus 18 answers) can be observed. As seven experts stated in their comments, possible misunderstandings or ambiguities in the statement resulted in a blank response.

Projection #13 (strategy and ecosystem) shows an ambiguity in form of a bimodal distribution in terms of the predicted occurrence time. Within #13, three experts expect legislative and political action or change in management to be a primary external driving force. One suggested that hospitals do not need individual strategies, but should focus on cross-clinic strategies to create “*super-hospitals*” (expert 37). Projection #15 (extinction of small hospitals in 2042) showed a clear bimodal distribution regarding the occurrence and desirability of the future extinction of small hospitals with regard to when the extinction will take place (47% 2042 and 32.4% later than 2042). 30.3% of experts perceive the projection as very undesirable and 27.3% as desirable. This leaves 42.4% as unsure. Statement #17 regarding gender-specific medicine with a doubling mode towards 2032 or 2037 (35.5% each) was also intensively discussed. Three experts addressed the availability and use of relevant data, and three experts lacked knowledge of gender-specific medicine, with an overall high desirability (mean=4.33).

In projection #18, which addresses data and health literacy of patients, experts showed disagreement regarding the predicted year of occurrence.

#### Cluster analysis statistical results

**Table 5:** Statistical overview of cluster comparison in four dimensions

			Probability Cluster 1	Probability Cluster 2	Impact Mean (C1)	Impact Mean (C2)	Desirability Mean (C1)	Desirability Mean (C2)
**#**	**- Projection Domain**	N C1/C2			IQR	EP (I)	IQR	EP (D)
	**Artificial Intelligence**	**29/7**						
1	Use of AI for treatment		62.2% 2 / 2032	57.1% 2 / 2032	3.79	3.14	4.48	3
2	Use of AI with multimodal data		65.2% 2 / 2032	71.4% 1 / 2027	4.03	2.71	4.66	4.0
3	Sensory monitoring		41.4% 1 / 2027	28% 1 28.6% 3	3.52	2.43	4.34	3.14
4	Use of AI in patient interaction as paid services		31% 2 31% n.a	42.9% n.a	2.45	1.43	3.03	1.29
5	Use of AI for systems medicine		55.2% 3 / 2037	57.1% n.a	3.66	1.43	4.07	n.a
	**Sustainability**	C3/C4 **22/9**	Probability Cluster 3	Probability Cluster 4				
6	Energy efficiency through AI		65.2% 3 / 2037	50% n.a/0	3.23	1.89	4.27	1.78
7	Energy efficient process design		43.5% 3 / 2037	30% 2 / 2032	3.32	2.11	4.59	3.56
8	Crisis resilience and robustness		30.4% n.a./0	60% n.a	3.45	2.78	4.64	4.33
9	Impact of climate change on patients in 2027^b^		3,59	3,22	3.91	3.67	1.36	1.56
	**Ecosystem**	C5/C6	Probability Cluster 5	Probability Cluster 6				
10	Changes in the structure of hospital services	**24/6**	58.3% 1 / 2027	50% 2 / 2032	3.58	3.50	3.75	2.83
11	Intersectoral connection via standards		54.2% 3 / 2037	33.3% 3 33.3% 5	4.00	4.50	4.42	4.67
12	Collaborative business model development with partners		41.7% 2 / 2032	50% n.a	3.42	2.17	3.92	3.00
13	Strategy & ecosystem		33.3% 3 / 2037	66.7% n.a	3.83	2.17	4.42	2.83
14	Decentralized medicine in 2032^b^		3.64	2.54	3.79	2.69	4.21	2.46
15	Near extinction of small hospitals in 2042^b^		4.21	3.62	4.57	3.54	3.57	1.92
16	Home treatment as is an established part of hospital services		73.3% 2 / 2032	42.9% 2 / 2032	3,93	3,69	4,43	4
	**Human-Centeredness**	C7/C8 **14/13**	Probability Cluster 7	Probability Cluster 8				
17	Gender specific medicine		40% 3 / 2037	50% 2 / 2032	2.57	3	3.93	4.38
18	Data & health literacy of patients		26.7% 2 / 2032	35.7% 4 / 2042	3.36	3.31	4.36	3.77
19	Change in activity profiles through the upgrading of tasks in 2032^b^		3,29	3.15	3.0	3.62	3.86	2.69
20	Development of new job professions and roles in 2037^b^		4.07	4.31	3.86	4.31	3,79	4,23
21	General practice shortage in 2032^b^		4.14	3.62	3.86	3.69	1.93	1.31
22	Growing staff shortage and workload increase in 2027^b^		4.36	4.15	4.57	4.46	1.36	1
23	Capacity overload due to ageing & multimorbidity in 2032^b^		3.36	3.54	4.0	3.92	2.0	1.92

Statements that are marked with (b) have been measured in fixed-time horizon and measured with mean. Other statements measured with mode frequency; in addition the mode probability on the scale 1–5 are displayed, as well as the percentage; n.a. no valid result (skipped question or lack of expertise)

## Abbreviation

AI: Artificial intelligence
