# Supplementary material for: Forecasting the future of smart hospitals: findings from a real-time delphi study
Source: BMC Health Serv Res. 2024 Nov 18;24:1421. doi: 10.1186/s12913-024-11895-z (PMC11572004; doi:10.1186/s12913-024-11895-z)
Supplement: Supplementary file 1 — Supplementary Material 1 [file 12913_2024_11895_MOESM1_ESM.docx]

**Supplementary Material 1:**English language version of the full questionnaire for the study
“Forecasting the future of smart hospitals – real-time Delphi study findings”

|  | # | DELPHI PROJECTION STATEMENT / QUESTION | Projection Domain |
| --- | --- | --- | --- |
| **Dimension 1: Artificial Intelligence** | 1 | Artificial Intelligence (AI) use in relevant processes of administration, diagnosis, and treatment contributes to more efficient, quality-improved, patient-oriented and better planned activities in the hospital. | Use of AI for treatment |
|  | 2 | In-hospital (AI-assisted) prediction, diagnosis, and treatment of disease is performed with multimodal input data. | Use of AI with multimodal data |
|  | 3 | AI-supported sensors record important parameters of inpatients and provide early warnings. | Sensory monitoring |
|  | 4 | AI-based solutions that serve direct patient interaction (e.g., voice assistants in the patient's room) are primarily established as individual health services/ add-ons in hospitals. | AI in patient interaction as  paid services |
|  | 5 | AI enables the application of systems medicine as part of personalized medicine. Hospitals use individualized medicine in standardized, scalable procedures to provide patients with precisely tailored and targeted treatments. | Use of AI for systems medicine |
| **Dimension 2: Sustainability** | 6 | The use of AI enables an energetically self-sufficient and efficient use of energy in the hospital. | Energy efficiency through AI |
|  | 7 | Treatment and administrative processes (especially AI-based processes) are designed to conserve resources and are climate-neutral in terms of energy. | Energy efficient process design |
|  | 8 | Hospitals are energy resilient and (AI-based) systems are secure against crises and attacks. | Crisis resilience |
|  | 9 | *In 2027, energy price fluctuations and resource scarcity lead to a significant increase in (treatment) case costs and insurance premiums for patients. | Impact of climate change on patients |
| **Dimension 3:  Ecosystems** | 10 | Market participants are changing the market position and service structure of hospitals with new, including AI-based offerings and services. | Changes in the structure of hospital services |
|  | 11 | Hospitals are intersectorally connected via uniform standards; they collect, exchange and process data, also for the use of AI solutions. | Intersectoral connection via standards |
|  | 12 | Maximum care hospitals develop digital and AI-supported solutions in sustainable business models with partners of their own ecosystem. | Collaborative business model development with partners |
|  | 13 | Maximum care hospitals in Germany have an elaborated, written, communicated, lived and agile future strategy. | Strategy & ecosystem |
|  | 14 | *In 2032, treatments will increasingly take place in several treatment centers at different locations (in the hospital, with specialists and in medical centers). | Decentralized medicine in 2032 |
|  | 15 | *In 2042, the number of hospitals has steadily decreased, so that predominantly only maximum care hospitals and specialist or private hospitals remain. | Near extinction of small hospitals in 2042 |
|  | 16 | Home treatment is an established part of the patient journey and hospital services. | Home treatment is an established part of hospital services |
| **Dimension 4:  Human-Centeredness** | 17 | Gender-specific medicine is well established in German hospitals. There is active work to resolve data bias and accumulate needed data to improve diagnoses and treatments. | Gender specific medicine |
|  | 18 | Patients act as facilitators of their own care and treatment process through existing data & health literacy. | Data & health literacy of patients |
|  | 19 | *In 2032, direct doctor-patient communication will no longer be necessary for preliminary discussions and standard diagnoses; any clarification will be provided by trained specialist staff. | Change in activity profiles through the upgrading of tasks in 2032 |
|  | 20 | *In 2037, new and changed roles and professions have entered hospitals: Due to new technologies (especially AI) and the increasing complexity of treatments and diseases, the task and activity profiles have become increasingly granular and specialized. | Development of new job professions and roles in 2037 |
|  | 21 | *In 2032, the number of general practitioners in private practice has decreased significantly, which leads to a deterioration in rural care. | General practice shortage in 2032 |
|  | 22 | * In 2027, growing staff shortages and rising case numbers will lead to even longer waiting times and inadequate care in hospitals. | Growing staff shortage and workload increase in 2027 |
|  | 23 | *In 2032, treatment cases are predominantly geriatric, chronic and multimorbid in nature, and at the same time the burden on hospital staff and processes reaches a peak. | Capacity overload due to aging & multimorbidity in 2032 |
| **Demographic  questions** | 24 | What corresponds to your age group? |  |
|  | 25 | How would you describe your gender? |  |
|  | 26 | How would you describe your current job position? |  |
|  | 27 | What sector are you currently working in? |  |
|  | 28 | What is the size of the organization that you are currently working in? |  |
|  | 29 | What is your level of education? |  |
|  | 30 | How many years of work experience do you have? |  |
|  | 31 | How would you rate your competences in AI, healthcare / medicine, sustainability, management of organizations, social science (please rank each competence level individually). |  |

Statements that are marked with a star (*) have been measured in fixed-time horizon.
